# Anterior chamber depth — a predictor of refractive outcomes after age-related cataract surgery

**DOI:** 10.1186/s12886-019-1144-8

**Published:** 2019-06-25

**Authors:** Xiaona Ning, Yuhuan Yang, Hong Yan, Jie Zhang

**Affiliations:** 1 Department of Ophthalmology, Xi’an No. 4 Hospital, Shaanxi Eye Hospital, Affiliated Guangren Hospital School of Medicine, Xi’an Jiaotong University, Xi’an, 710004 Shaanxi Province China; 2 Department of Ophthalmology, Tangdu Hospital, Fourth Military Medical University, Xi’an, 710038 Shaanxi Province China

**Keywords:** Cataract, Pentacam HR, Anterior chamber depth, Axial length, Refractive errors

## Abstract

**Background:**

Anterior chamber depth (ACD) is becoming a hot topic and plays an important role in correcting the refractive errors (REs) after cataract surgery. The aim of this study was to assess the ACD changes and their relationship with the REs after phacoemulsification and intraocular lens (IOL) implantation in patients with age-related cataracts.

**Methods:**

One hundred forty-five eyes of 125 age-related cataract patients from the Department of Ophthalmology, Tangdu Hospital, China, were recruited. IOL Master was used for axial length (AL) and the IOL power calculation measurements, and the Pentacam HR device was used for the ACD and lens thickness (LT) measurements. Every patient underwent uncomplicated phacoemulsification by a single surgeon using a single technique. Postoperative refraction results were obtained at 1 month. The appropriate formula used for the IOL power calculation was chosen depending on the AL, specifically the Hoffer Q (AL < 22.0 mm), SRK/T (22.0 mm ≤ AL ≤ 30.0 mm), and Haigis (AL > 30.0 mm) formulas.

**Results:**

The postoperative ACD was deepened and tended to stabilize gradually after 2 weeks. A concurrent hyperopic shift (0.57 ± 0.47 D) was observed when the change in the ACD was less than 1.65 mm, whereas a myopic shift (− 0.18 ± 0.62 D) occurred contrarily, and the difference between the two groups was statistically significant (*P* < 0.0001). The change in ACD was significantly larger in the shallow anterior chamber (1.92 ± 0.40 mm) than in the deep chamber (1.33 ± 0.42 mm) (*P* < 0.0001). Similarly, the change in ACD was larger in the short AL (2.12 ± 0.37 mm) than in the long AL (1.32 ± 0.49 mm). The postoperative ACD and refractive changes were correlated with the preoperative ACD and AL (*P* < 0.0001), respectively. Two regression formulas were proposed: postoperative ACD = 3.524 + 0.294 × preoperative ACD and postoperative ACD = 3.361 + 0.228× (preoperative ACD + 1/2 LT).

**Conclusions:**

The results of this study showed that the ACD deepened and was associated with a concurrent RE after cataract surgery. Postoperative changes in the ACD were related to the preoperative ACD and AL, which determined the refraction status and visual quality. The regression formula of the postoperative ACD could provide a theoretical basis for predicting refractive errors in the clinic.

## Background

Cataract is one of the main reasons for visual impairment or loss worldwide [1], and the number of patients visually impaired was estimated by the WHO to be 95 million in 2014 [2]. Surgery with phacoemulsification and implantation of a posterior chamber intraocular lens (IOL) is the only effective treatment for managing cataract [3, 4]. At present, with the rapid development of surgical and IOL technology, the goal of cataract surgery has changed from simply blindness relief simply to accurate refractive correction [5]. Therefore, refractive errors (REs) after cataract surgery resulting from IOL is one of the most urgent problems to be solved.

The postoperative RE is affected by many factors. The primary and most common sources include the measurement of the ocular parameters (keratometry, axial length (AL), lens thickness (LT), the selection of IOL calculation formula, the position of IOL implantation and so on [6], which determine the postoperative visual acuity of patients. To minimize postoperative RE, a comprehensive analysis of the above-mentioned biological characteristics should be performed [7]. Even though all these measurements are carried out, refractive corrections postoperatively are often needed, and the RE of 9 to 20% patients is more than 1 dioptre (D) [8]. Currently, it is believed that among the factors affecting postoperative visual function, the stability of the IOL postoperative position has recently been considered to be one of the key factors that can be represented by effective lens position (ELP) [9, 10]. This means that improvement in refractive outcome requires better methods for predicting ELP.

The anterior chamber depth (ACD) refers to the distance between the anterior surface of the cornea and the anterior surface of the lens, which is an indicator of the axial position of the IOL (so-called ELP) postoperatively [11]. ELP could predict that forward deviation of the IOL leads to myopia, conversely leading to hyperopia [12]. Olsen found that 42% of IOL power prediction errors were caused by incorrect estimation of the postoperative ACD [13], which means that considering ACD into the calculation of the IOL power is probably an effective method to reduce postoperative errors. Among the existing calculation formulas of IOL power, SRK/T only measures the AL and keratometry, while the newer formulas (such as Haigis, Olsen, Holladay 2) often consider the preoperative ACD measurement, which is helpful in predicting the postoperative ELP and RE [14, 15]. Therefore, ACD plays an important role in correcting the postoperative RE, which deserves further study. However, the way to access and predict postoperative ACD still remains unclear.

The purpose of this study was to assess the clinical and biometric parameters, especially ACD, associated with postoperative RE after uneventful cataract surgery. For this purpose, we collected the clinical data of patients and followed up at least 1 month to analyse how the ACD influenced RE, to identify the key influencing factors and devise a way to predict postoperative ACD. These results are meaningful and may offer a better method of ELP prediction to help patients gain better vision quality after cataract surgery.

## Methods

### Study design and patient selection

In a retrospective study from July 2015 to January 2017, data from a total of 125 patients with age-related cataracts (145 eyes) referred to Tangdu Hospital affiliated with the Fourth Military Medical University in Xi’an, Shaanxi, were extracted. All research and measurements followed the tenets of the Declaration of Helsinki, and the protocol was reviewed and approved by the Ethics Committee of Tangdu Hospital (TDLL 201506–05). Informed consent was obtained before the research from all patients. Inclusion criteria were patients with age-related cataracts who underwent phacoemulsification combined with in-the-bag IOL implantation without any operative accident. The exclusion criteria included a history of diseases affecting refraction, such as lacrimal duct diseases, glaucoma, corneal pannus, keratoconus, corneal oedema, pterygium, intraocular surgery; a history of fundus diseases, such as retinal detachment, retinal splitting, choroidal neovascularization, and vitreous haemorrhage.

### Surgical procedure

Patients underwent a comprehensive physical examination and eye examination before surgery. Preoperatively, the clinical examination included optometry, intraocular pressure (IOP), fundus photography, anterior segment photography, B-ultrasound, OCT, corneal endothelial count, IOL master, visual electrophysiology, Pentacam, and routine blood tests. To prepare the eyes before surgery, patients were treated with bilateral levofloxacin eye drops 0.5% (Cravit, Santen, Japan) four times per day for 3 days to prevent infection. Then, patients were given tropicamide phenylephrine eye drops 0.5% (Mydrin-P, Santen, Japan) to maximize pupil dilation beginning a half an hour before surgery. During the surgery, the same micro-incision phaco machine (Bausch & Lomb, USA) was used by the same senior surgeon (Hong Yan). First, patients were anaesthetized topically with oxybuprocaine hydrochloride eye drops 1.0% (Benoxil, Santen, Japan), and then a 3.0 mm clear corneal incision was made at the steep meridian. A soft-shell technique was used to protect the corneal endothelium and maintain anterior chamber space. A centred circular continuous capsulorhexis with a diameter of 5.5 mm was made with capsulorhexis forceps. Then, thorough hydrodissection was performed to rotate the nucleus freely, and nuclear fracturing was performed using the stop-chop method. After phacoemulsification, the posterior chamber IOL was implanted into the capsular. Different types of IOL were chosen depending on the different requirements of the patients. After surgery, patients were treated with levofloxacin eye drops 0.5% (Cravit, Santen, Japan) and prednisolone acetate ophthalmic suspension 1% (Pred Forte, Allergan, Ireland) 4 times per day for 2 weeks after surgery. The postoperative clinical examination included optometry, IOP, and Pentacam 2 days, 2 weeks and 1 month after surgery.

### Data collection

A questionnaire that included data about age, sex, AL, LT and ACD was completed for each patient based on their records. The ACD and AL were extracted from the biometry reports. The AL was measured with IOL Master 500 (Carl Zeiss, Germany), which was repeated five times for each patient, and the average AL was calculated by the machine. The LT and ACD were measured with a Pentacam HR Scheimpflug device 70,900 (OCULUS, Germany), which was calculated automatically by the machine. All these measurements were carried out by a trained medical worker. The results of Pentacam were extracted only when the quality of the examination showed “OK”. The ACD we adopted here was determined based on the central inner corneal surface, perpendicular to the corneal surface to the most anteriorly visible part of the IOL. The RE calculated as the arithmetic deviation from the preoperative target refraction. The absolute refractive error refers to the absolute deviation between postoperative RE and preoperatively expected RE.

### Classification and measurements

An AL < 22.0 mm was considered short sighted, 22.0 mm ≤ AL ≤ 26.0 mm was a normal AL and an AL > 26.0 mm was considered long sighted. For the ACD, we considered an ACD < 2.66 mm as low depth and an ACD ≥ 2.66 mm as high depth (ACD = 2.66 mm was the median of the whole data). For the change in ACD, we separated the data into two groups: the A group (change of ACD < 1.65 mm) and the B group (change of ACD ≥ 1.65 mm) (change of ACD = 1.65 mm was the median of the whole data). IOL Master was performed to determine an optimization formula for the IOL choice based on the AL. The IOL power was decided by obtaining the postoperative refraction at approximately − 0.50 dioptre (D) with Hoffer Q (AL < 22.0 mm), SRK/T (22.0 mm ≤ AL ≤ 30.0 mm), and Haigis (AL > 30.0 mm) formulas. Preoperative best-corrected visual acuity (BCVA) was recorded.

### Statistical analysis

Data were analysed using SPSS version 19.0 (SPSS Inc., Chicago, IL, USA). One-way ANOVA was used to analyse differences between three groups. The independent-samples t-test was used to compare quantitative data between two groups when satisfying bivariate normal distribution, and if not, the Mann-Whitney U test was used. The paired t-test was used to compare the preoperative and postoperative results of the same patient. The Pearson correlation coefficient was calculated to analyse the relationship between the quantitative data that satisfied the bivariate normal distribution. The Spearman correlation coefficient was calculated to analyse the univariate variables that satisfied the normal distribution. On the premise that the regression diagnostics were applied to test the validity of the linear regression models and the equations derived, the linear regression model was used to obtain a mathematical model to estimate postoperative ACD using preoperative ACD and LT. All the data are displayed as the mean ± standard deviation. A *p*-value of less than 0.05 was considered statistically significant.

## Results

For the 125 cataract patients included whose Pentacam data were complete at 2 days, 2 weeks and 1 month postoperative, 145 cases (eyes) were studied overall, and 69 patients were men (47.6%). The mean age of the patients was 67.7 ± 11.5 years. Of the patients whose Pentacam and optometry data were complete both preoperatively and 1 month postoperatively, 123 cases (eyes) were studied due to loss to follow-up, of which 60 cases were men (48.8%), and their mean age was 67.8 ± 10.1 years. Regrettably, of the 123 cases, only 55 cases of LT were successfully measured preoperatively and 1 month postoperatively; 29 cases were men (52.7%), and the mean age was 69.6 ± 8.5 years. All patients underwent uneventful surgeries without intraoperative or postoperative complications.

### Postoperative ACD among different visits

**Fig. 1:**
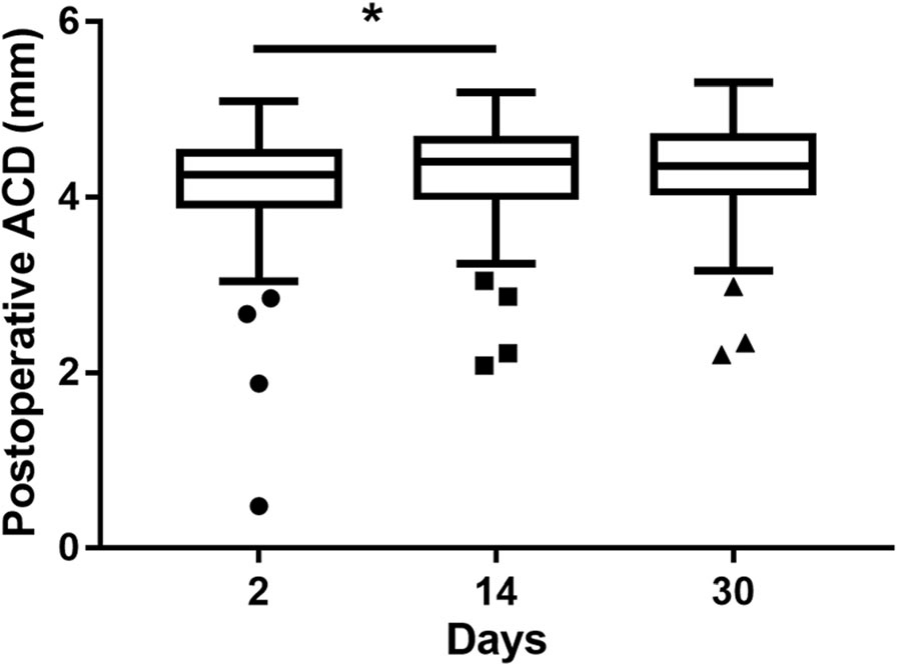
Comparison of the postoperative ACD from different visits. ACD: anterior chamber depth. (*P = 0.016, *n* = 145)

At 2 days, 2 weeks and 1 month postoperatively, the ACD was 4.16 ± 0.59 mm, 4.32 ± 0.52 mm, and 4.31 ± 0.53 mm, respectively. There was a significant difference between 2 days and 2 weeks (*P* = 0.016) postoperatively, but there was no difference between 2 weeks and 1 month (*P* = 0.899) postoperatively. This indicated that the ACD tended to stabilize gradually 2 weeks postoperatively (Fig. 1). After phacoemulsification with a clear corneal incision, the ACD is relatively stable 2 weeks later. Therefore, we chose the postoperative 1 month period as the time point for collecting all the following data.

### Influence of ACD on refractive errors after cataract surgery

The refractive errors of the A group (change in ACD <1.65 mm) and the B group (change in ACD >1.65 mm) were 0.57 ± 0.47D and − 0.18 ± 0.62D, respectively. The absolute refractive errors of the A and B groups were 0.60 ± 0.43D and 0.44 ± 0.48D, respectively. As shown in Table 1, there was a significant difference in the refractive errors between the two groups (*P* < 0.001) but little difference in the absolute refractive errors (*P* = 0.049). This indicated that a hyperopia shift of refractive errors would often occur when the ACD had a less amount of change after cataract surgery, whereas a myopic shift was related to a greater change in the ACD.

**Table 1:** Comparison of refractive errors according to the classification of the change in anterior chamber depth

Changes in ACD	N (eyes)	RE (D)	Absolute RE (D)
A group (< 1.65 mm)	61	0.57 ± 0.47	0.60 ± 0.43
B group (≥ 1.65 mm)	62	− 0.18 ± 0.62	0.44 ± 0.48
*t*-value		−7.44	−1.99
*P*-value		0.000	0.049

*ACD* anterior chamber depth, *D* dioptre, *RE* refractive error

### Impact of the preoperative anterior chamber depth and axial length on the change of the postoperative anterior chamber depth and refractive errors

We regarded a preoperative ACD < 2.66 mm as the A group and an ACD ≥ 2.66 mm as the B group. As shown in Table 2, the changes in the postoperative ACD were 1.92 ± 0.40 mm and 1.33 ± 0.42 mm, which were significantly different between the two groups (*P* < 0.001). The postoperative refractive errors were − 0.15 ± 0.65D and 0.53 ± 0.49D, which were significantly different between the two groups (*P* < 0.001). However, there was no significant difference in the absolute refractive errors. The lower preoperative anterior chamber group varied more in the postoperative ACD.

**Table 2:** Comparison of the change in postoperative anterior chamber depth and refractive errors according to the classification of preoperative anterior chamber depth

Preoperative ACD	N (eyes)	Changes in ACD (mm)	RE (D)	Absolute RE (D)
A (< 2.66 mm)	61	1.92 ± 0.40	−0.15 ± 0.65	0.44 ± 0.49
B (≥2.66 mm)	62	1.33 ± 0.42	0.53 ± 0.49	0.60 ± 0.42
*t*-value		−7.88	6.55	1.77
*P*-value		0.000	0.000	0.079

*ACD* anterior chamber depth, *D* dioptre, *RE* refractive error

With the classification of preoperative AL, we regarded preoperative AL < 22.0 mm as the A group, 22.0 mm ≤ AL ≤ 26.0 mm as the B group, and AL > 26.0 mm as the C group. As shown in Table 3, the changes in the postoperative ACD were 2.12 ± 0.37 mm, 1.59 ± 0.44 mm and 1.32 ± 0.49 mm for the three groups, respectively. The postoperative refractive errors were − 0.55 ± 0.66D, 0.19 ± 0.47D and 0.77 ± 0.53D for the three groups, respectively. The postoperative absolute refractive errors were 0.59 ± 0.62D, 0.39 ± 0.32D and 0.79 ± 0.50D for the three groups, respectively. The overall change in ACD, refractive errors and absolute refractive errors were significantly different in the short AL compared to the normal AL (*P* < 0.05) and in the long AL (*P* < 0.01). These data indicated that the postoperative change in the ACD was related to the preoperative ACD and AL.

**Table 3:** Comparison of the change in postoperative anterior chamber depth and refractive errors according to the classification of preoperative axial length

AL (mm)	N (eyes)	Changes in ACD (mm)	RE (D)	Absolute RE (D)
A (< 22.0)	22	2.12 ± 0.37^a^	−0.55 ± 0.66^a^	0.59 ± 0.62^a^
B (22.0–26.0)	72	1.59 ± 0.44	0.19 ± 0.47	0.39 ± 0.32
C (> 26.0)	29	1.32 ± 0.49^b^	0.77 ± 0.53^b^	0.79 ± 0.50^b^
*P*-value^a^		0.000	0.000	0.040
*P*-value^b^		0.008	0.000	0.000

^a^Comparison of A and B group

^b^Comparison of B and C group

*ACD* anterior chamber depth, *D* dioptre, *RE* refractive error

### Correlation analysis of the postoperative refractive errors

**Fig. 2:**
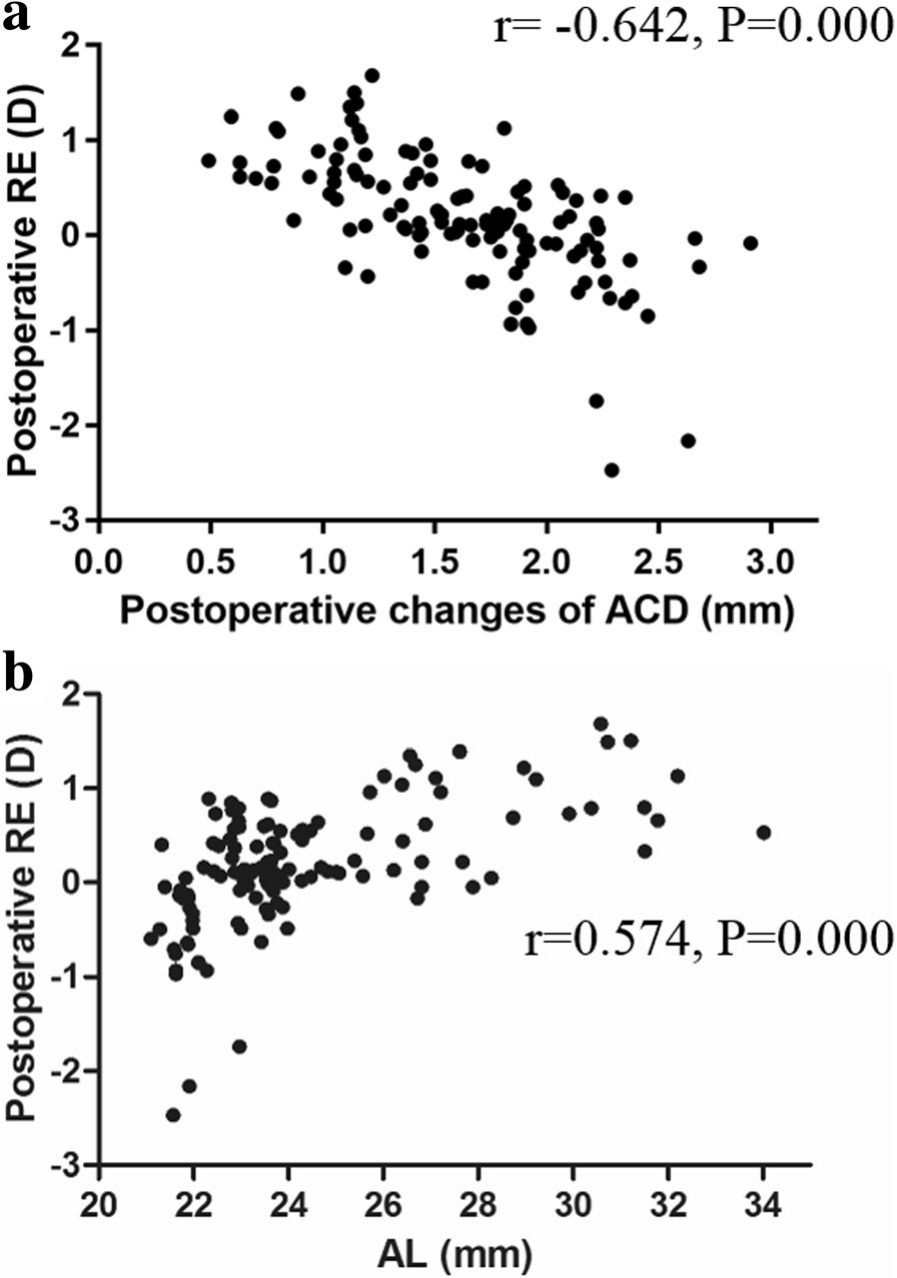
Correlation analysis of the postoperative refractive errors. **a** The scatter plot of the relationship between the postoperative change in ACD and the postoperative RE. **b** The scatter plot of the relationship between AL and postoperative RE. (*n* = 123, *P* < 0.001). ACD: anterior chamber depth; AL: axial length; D: dioptre; RE: refractive errors

As shown in Table 4, the Pearson correlation tests showed that there was a direct (linear) and positive relationship between the preoperative ACD and the postoperative refractive errors (r = 0.541; *P* < 0.001) and between the AL and the refractive errors (r = 0.574; *P* < 0.001). Regarding the postoperative change in ACD, postoperative change of ACD/ preoperative ACD, postoperative change of ACD/AL and LT/ preoperative ACD, there was a direct (linear) and negative relationship between these and the postoperative refractive errors (r = − 0.642, − 0.607, − 0.698, − 0.306, respectively; *P* < 0.001). The postoperative ACD and LT were not correlated with the postoperative refractive errors (*P* > 0.05). The scatter plot in Fig. 2a also indicated that the postoperative change in ACD was negatively correlated with the postoperative refractive errors, which means that the postoperative refractive errors tended towards a myopic shift when the ACD changed a large amount (Fig. 2a). The scatter plot in Fig. 2b also proved that the AL was positively correlated with the postoperative refractive errors, which means that the postoperative refractive errors tended towards a hyperopia shift when the AL was longer (Fig. 2b).

**Table 4:** Correlation analysis of refractive errors

Correlative factors	N (eyes)	*r*	*P*-value
Preoperative ACD	123	0.541	0.000
Postoperative ACD	123	–	0.178
Postoperative changes in ACD	123	−0.642	0.000
Postoperative changes in ACD / Preoperative ACD	123	−0.607	0.000
Postoperative changes in ACD /AL	123	−0.698	0.000
AL	123	0.574	0.000
LT	55	–	0.595
LT / Preoperative ACD	55	−0.306	0.023

“—” means there is no correlation

*ACD* anterior chamber depth, *AL* axial length, *LT* lens thickness, *r* correlation coefficient

### Correlation analysis of the change of the postoperative anterior chamber depth

**Fig. 3:**
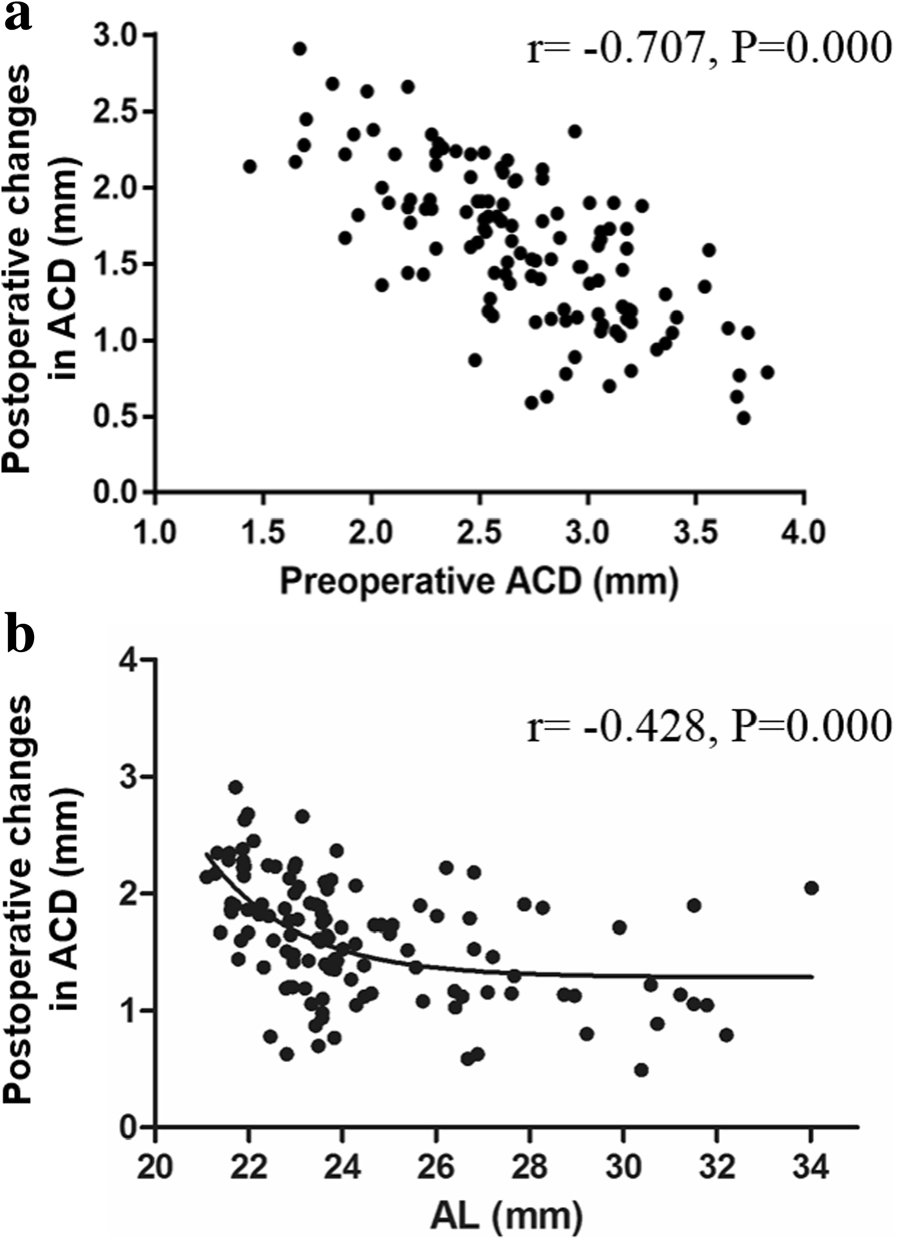
Correlation analysis of the postoperative changes in ACD. **a** The scatter plot of the relationship between the preoperative ACD and the postoperative change in ACD. **b** The scatter plot of the relationship between the AL and the postoperative change in ACD. (*n* = 123, *P* < 0.001). ACD: anterior chamber depth; AL: axial length

As shown in Table 5, the Pearson correlation tests showed that there was a direct (linear) and negative relationship between the preoperative ACD and postoperative change in ACD (r = − 0.707; *P* < 0.001) and between the AL and the refractive errors (r = − 0.428; *P* < 0.001). The postoperative ACD was directly (linear) and positively correlated with the postoperative change in ACD (r = 0.368, *P* < 0.001), as it was with the LT/ preoperative ACD (r = 0.674, *P* < 0.001). The LT was not correlated with the postoperative change in ACD (*P* > 0.05). The scatter plot in Fig. 3a proved that the preoperative ACD was negatively correlated with the postoperative change in ACD, which means that the postoperative ACD changed more when the preoperative ACD was shallower (Fig. 3a). The scatter plot in Fig. 3b proved that the AL was negatively correlated with the postoperative change in ACD, which means that the postoperative change in the ACD was greater when the AL was longer (Fig. 3b).

**Table 5:** Correlation analysis of the change in postoperative anterior chamber depth

Correlative factors	N (eyes)	*r*	*P*-value
Preoperative ACD	123	−0.707	0.000
Postoperative ACD	123	0.368	0.000
AL	123	−0.428	0.000
LT	55	–	0.211
LT / Preoperative ACD	55	0.674	0.000

“—” means there is no correlation

*ACD* anterior chamber depth, *AL* axial length, *LT* lens thickness, *r* correlation coefficient

### Prediction of the postoperative anterior chamber depth

**Fig. 4:**
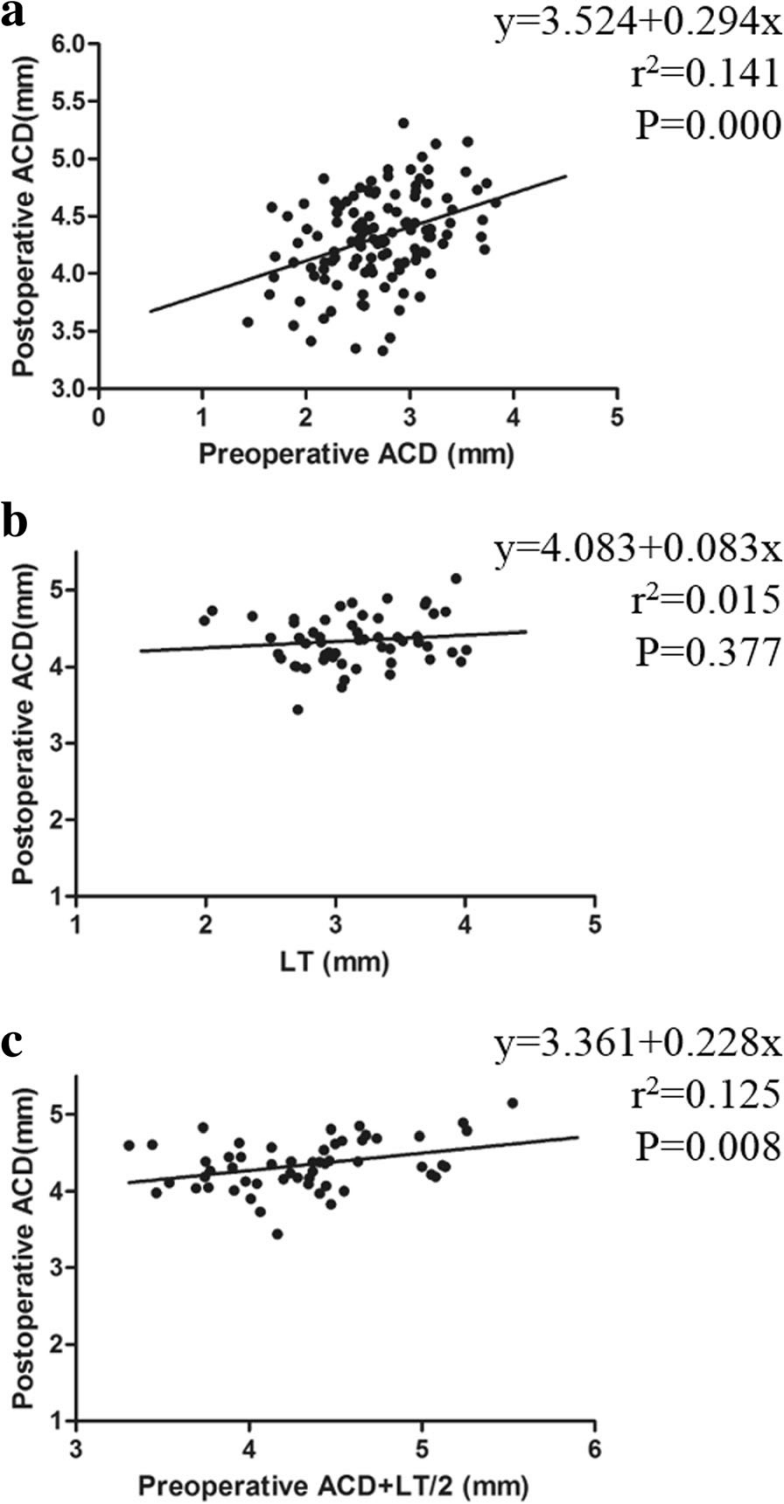
Correlation analysis of the postoperative ACD. **a** The scatter plot of the postoperative ACD and the preoperative ACD. (*n* = 123, *P* < 0.001); **b** The scatter plot of the postoperative ACD and LT. (*n* = 55, *P* > 0.05); **c** The scatter plot of the postoperative ACD and preoperative ACD +1/2 LT. (*n* = 55, *P* < 0.01). ACD: anterior chamber depth; LT: lens thickness

A linear regression model was used to assess the relationship between the postoperative ACD, preoperative ACD and LT. The following result was obtained: postoperative ACD = 3.524 + 0.294 × preoperative ACD (Fig. 4a); postoperative ACD = 3.361 + 0.228× (preoperative ACD + 1/2 LT) (Fig. 4c); postoperative ACD was not correlated with the LT (*P* > 0.05, Fig. 4b).

## Discussion

Cataracts remain the leading cause of vision loss, and their prevalence increases with age. Cataract extraction and IOL implantation is an effective treatment, and the one most commonly used. With the development of technology, the purpose of surgery is not only to gain vision but also to see clearly and comfortably, which means allowing patients to have a better refractive status and visual experience [16]. Postoperative emmetropia is the major determining factor for patient satisfaction after cataract surgery. The ACD is an index that reflects the effective position of the IOL, which means that the prediction errors of the postoperative ACD will lead to a myopia or hyperopia shift after cataract surgery [17]. Therefore, ACD plays an important role in predicting postoperative RE after cataract surgery.

Our study demonstrated that cataract surgery deepens the ACD and that it tends to stabilize gradually 2 weeks after surgery. The change in the ACD derived from cataract surgery had an impact on refractive errors; a hyperopic shift would often occur when the change in the ACD was smaller, whereas a myopic shift was related to a larger change in the ACD. Next, we discussed the factors that influence changes in the ACD and found that the preoperative ACD and AL were related to changes in the ACD. Then, we performed a correlation analysis of the postoperative RE and the change in the ACD with some possible factors. The postoperative RE was negatively correlated with the postoperative changes in the ACD and positively correlated with the AL. Similarly, the postoperative changes in the ACD were negatively correlated with the preoperative ACD and AL. Furthermore, we proposed possible formulas for predicting the postoperative ACD: postoperative ACD = 3.524 + 0.294 × preoperative ACD and postoperative ACD = 3.361 + 0.228× (preoperative ACD + 1/2 LT). The possible reasons for our results are as follows.

There are different opinions about the period of time necessary to achieve postoperative refractive stability. The stability occurs between 2 weeks [18] and 6 weeks. We considered 1 month to be an acceptable period of time for postoperative refractive stability to develop. It has been reported that for each additional 1 mm change in the ACD, there is at least an extra 0.32D of refractive shift after cataract surgery [13]. Although the postoperative ACD, which represented the IOL effective position, had been considered in the formula of the IOL power calculation, it was still affected by other factors causing the postoperative refractive status to be a myopic or hyperopic shift [19–23]. Most of the scholars and researchers agree that a postoperative refractive shift occurred and have studied this [24–26]; however, there has been no consistent opinion about the degree and the trend of this refractive shift. Our results indicated that a hyperopic or myopic shift often occurs with different degrees of change in the ACD.

Regarding the degree of change in the ACD after cataract surgery, some studies have reported that the depth of the preoperative anterior chamber is related to the postoperative ACD change [27]. Our study also proved that the changes in the ACD in the shallow and deep anterior chamber were 1.92 ± 0.40 mm and 1.32 ± 0.42 mm, respectively, which implied that the postoperative change in the ACD was negatively related to the preoperative ACD. In addition, Muzyka-Wozniak [28] evaluated the postoperative ACD after phacoemulsification in eyes with a short or long axial length and found that the relative change in the ACD was larger in short eyes (57%) than in normal eyes (44%) or long eyes (42%) (*P* < 0.017), which means that the AL influenced changes on the postoperative ACD. This finding was consistent with our study, which indicated that the changes in the ACD with short, normal and long AL were 2.12 ± 0.37 mm, 1.59 ± 0.44 mm and 1.32 ± 0.49 mm, respectively, which indicated that the postoperative change in the ACD was also negatively related to the preoperative ACD. We next conducted a correlation analysis and proved this further. Taken together, the shallower the anterior chamber and the shorter the AL were, the greater were the changes in the ACD. Therefore, the refractive status of a patient with a shallow anterior chamber and a short AL would trend towards a myopic shift, conversely, a deep anterior chamber and long AL would move toward a hyperopic shift. We speculated that this phenomenon may result from a slight deviation in the ability the IOL Master formula to predict the IOL power. Because predicted postoperative ACD value of the patient with a shallow anterior chamber was larger and that of the patient with a long AL was smaller, the actual postoperative IOL, and the preoperative estimated position were not consistent. Thus, it raised the myopia or hyperopia refractive errors shift postoperatively. However, some studies differ from our results [29], and further studies will be needed.

The change in refractive status after cataract surgery is closely related to postoperative ELP. For the prediction of the postoperative ACD, Ucakhan et al. [30] noted an obvious backward shift of the iris at the beginning of the postoperative period, especially in eyes with very shallow anterior chambers. Pereira et al. [27] also found that the distance between the iris and IOL was increased over a period after cataract surgery, and the same result was observed by OCT in the anterior segment [31]. Although it is difficult to estimate the ELP after cataract surgery, researchers are still seeking a solution [9, 32, 33]. A recent study showed that the preoperative ACD demonstrated the greatest influence on the IOL calculation formulas, and the postoperative change in the ACD correlated significantly with errors of third generation formulas according to a simulated ACD [34]. In our study, we analysed the correlation between the postoperative and preoperative ACD and the results showed that postoperative ACD was positively correlated with the preoperative ACD and “preoperative ACD+1/2 LT”, but there was no relationship with the LT. We considered the reason for the lack of correlation between the postoperative ACD and LT was that the expansion degree of the lens and the squeezing degree to the anterior chambers were different among the patients, which led to greater uncertainty about the IOL position.

The limitations of this study were that three different formulas that depended on a different AL to calculate the IOL were applied in this manuscript, which may itself induce a RE. The sample size was also small when we divided the subjects into three groups, especially for the AL < 22.00 mm (*n* = 22) and the AL > 26.0 mm (*n* = 29) groups. Some of the patients were lost to follow-up because they could not adhere to the schedule, so we merely analysed the data from 1 month after surgery. Further studies are needed with a larger population, a longer follow-up period, and a multivariate regression of potential risk factors with one widely useful formula that can be adjusted to all AL.

## Conclusions

In conclusion, our preliminary results suggested that the ACD played an important role in predicting postoperative RE after cataract surgery. A hyperopia shift would often occur when the change in the ACD was smaller, whereas a myopic shift was related to a larger change in the ACD, which determined the refraction status and visual quality. In addition, the regression formula of the ACD could provide a theoretical basis for predicting refractive errors in the clinic. Further prospective studies are needed to identify a widely useful formula for the clinical guidance of age-related cataract surgery.

## Data Availability

The datasets used and analysed during the current study are available from the corresponding author on reasonable request.

## Abbreviations

ACD: Anterior chamber depth
AL: Axial length
IOL: Intraocular lens
LT: Lens thickness
RE: Refractive errors
